# Preliminary Evaluation of the Gut Microbiota Modulatory Potential of Malaysian Kefir Water in Ageing Mice

**DOI:** 10.3390/foods14223851

**Published:** 2025-11-11

**Authors:** Muganti Rajah Kumar, Aaron Opoku Amankwaa, Nurulain Syahirah Razali, Nurul Elyani Mohamad, Melati Khalid, Janna Ong Abdullah, Mas Jaffri Masarudin, Mohd. Azuraidi Osman, Nik Mohd Afizan Nik Abd Rahman, Noorjahan Banu Alitheen

**Affiliations:** 1 Department of Cell and Molecular Biology, Faculty of Biotechnology and Biomolecular Sciences, Universiti Putra Malaysia, UPM, Serdang 43400, Selangor, Malaysia; drmuganti@gmail.com (M.R.K.); nurulainsyahirahrs@gmail.com (N.S.R.); janna@upm.edu.my (J.O.A.); masjaffri@upm.edu.my (M.J.M.); azuraidi@upm.edu.my (M.A.O.); m.afizan@upm.edu.my (N.M.A.N.A.R.); 2 School of Geography and Natural Sciences, Northumbria University, Ellison Building, Newcastle upon Tyne NE1 8ST, UK; 3 Biotechnology Research Institute, Universiti Malaysia Sabah, Kota Kinabalu 88400, Sabah, Malaysia; elyani.mohamad@ums.edu.my; 4 Department of Biomedical Sciences, Faculty of Medicine and Health Sciences, Universiti Putra Malaysia, UPM, Serdang 43400, Selangor, Malaysia; melati@upm.edu.my; 5 UPM-MAKNA Cancer Research Laboratory, Institute of Bioscience, Universiti Putra Malaysia, UPM, Serdang 43400, Selangor, Malaysia

**Keywords:** kefir water, probiotic, ageing, D-galactose, dysbiosis, gut microbiota, *Lactobacillus*

## Abstract

Ageing is often accompanied by gut microbiota alterations that contribute to dysbiosis—a recognised hallmark of ageing and a risk factor for neurodegenerative diseases. Probiotic interventions offer a promising approach to restore microbial homeostasis. This preliminary study explored the potential modulatory effects of Malaysian kefir water, a *Lactobacillus*-enriched fermented beverage with previously reported antioxidant and neuroprotective properties in D-galactose-induced ageing mice. Kefir water was administered as both a pre-treatment and co-treatment, and gut microbiota changes were assessed using 16S rRNA metagenomic sequencing of faecal samples. Alpha and beta diversity analyses showed a stable microbial diversity across treatments. However, preliminary descriptive trends suggested that kefir water may influence specific bacterial populations. Increases were observed in *Muribaculaceae* and *Lactobacillaceae*, along with apparent decreases in *Lachnospiraceae* and *Prevotellaceae*. Both kefir treatments tended to increase the abundance of *Ligilactobacillus*, with the co-treatment group appearing to restore the *Firmicutes*/*Bacteroidota* ratio toward control levels, while the pre-treatment group showed a tendency to further reduce this ratio. Collectively, these findings provide preliminary indications that kefir water may hold potential as a dietary approach to modulate gut microbial changes associated with ageing. However, confirmation through studies with larger sample sizes and broader analytical coverage is necessary to substantiate these initial observations.

## 1. Introduction

According to the World Health Organization (WHO), by 2030, one in six people globally will be aged 60 years or older. Between 2020 and 2050, the global population aged ≥ 60 years is expected to double to 2.1 billion, while those aged ≥80 years will triple to reach 426 million [1]. Ageing remains the most significant risk factor for neurodegenerative diseases (ND), particularly Alzheimer’s disease (AD), which is characterised by progressive neuronal loss leading to cognitive and memory decline. Importantly, ageing is closely linked to the disruption of host–microbiota homeostasis, resulting in gut dysbiosis—an imbalance that contributes to systemic inflammation and the onset of age-associated disorders, including ND and AD. López-Otín et al. [2] recently expanded their framework of the nine hallmarks of ageing to include intestinal dysbiosis, highlighting its emerging role as a key contributor to age-related physiological decline. This addition underscores the central importance of the gut–brain axis in ageing biology and neurodegenerative disease development.

The human gut harbours trillions of microorganisms, including bacteria, fungi, archaea, and viruses—collectively referred to as the gut microbiome. This diverse community encodes over three million genes, vastly exceeding the 23,000 genes within the human genome, and comprises approximately 2000 bacterial species [3]. The gut also contains an intricate network of neurons embedded in the gastrointestinal wall, known as the enteric nervous system (ENS), which houses about 168 million neurons, a number comparable to the 222 million neurons found in the spinal cord [4]. Due to this extensive neuronal network, the gut is deeply involved in maintaining central nervous system (CNS) health, influencing cognition, memory, mood, and social behaviour, as well as modulating the ENS, autonomic nervous system, and hypothalamic–pituitary–adrenal (HPA) axis [5]. Consequently, the gut is often referred to as the “second brain”. Communication between the gut and the brain occurs bidirectionally through the gut–brain axis, which integrates neural (vagus nerve), endocrine, immune, and circulatory pathways, alongside lymphatic and glymphatic systems. These pathways enable the gut microbiome to modulate brain activity, while the brain, in turn, regulates intestinal motility, permeability, and secretory responses [5].

Gut microbiome composition undergoes dynamic changes with ageing and has been closely associated with the onset of ageing-related diseases. These shifts are influenced by genetic background, lifestyle, diet, environmental exposure, and geographical location, contributing to wide inter-individual variation. In general, ageing is accompanied by a decline in beneficial commensal taxa such as *Bifidobacterium*, *Faecalibacterium*, *Prevotella*, *Lachnospira*, *Ruminococcus*, *Eubacterium rectale*, and *Coprococcus*, which are gradually replaced by other commensals such as *Butyricimonas*, *Akkermansia*, *Christensenellaceae*, *Odoribacter*, and *Butyricicoccus*, and opportunistic pathobionts such as *Streptococcus*, *Eggerthella*, *Enterobacteriaceae*, *Bilophila*, and *Fusobacteria* [6,7]. For instance, Lee et al. [8] reported a higher abundance of *Lactobacillus* in younger individuals compared to the elderly, who displayed increased levels of *Escherichia*, *Bacteroides*, and *Clostridium*. Similarly, Sepp et al. [9] found a decline in butyrate-producing bacteria such as *Faecalibacterium prausnitzii* and *Lachnospiraceae* in centenarians, consistent with studies from Chinese, Korean, and Italian cohorts [10,11,12]. The loss of butyrate-producing bacteria in ageing may exacerbate metabolic dysfunction, given butyrate’s role in appetite regulation, insulin sensitivity, and maintaining a healthy gut barrier.

Emerging evidence indicates that gut dysbiosis contributes to neurodegenerative processes through the production of microbial metabolites that promote amyloid-beta aggregation, tau hyperphosphorylation, and neuroinflammation. For instance, Honarpisheh et al. [13] demonstrated that dysbiosis in AD mouse models enhances amyloid-beta accumulation, whereas Kim et al. [14] reported that faecal microbiota transplantation from healthy mice reduced amyloid and tau pathology, improved cognition, and decreased systemic inflammation. Similarly, transplanting an aged microbiome into young germ-free mice induced inflammation, increased gut permeability, and decreased *Akkermansia* abundance, exhibiting traits of inflamm-ageing [15]. These findings underscore the role of the aged gut microbiome as a potential driver of systemic and neuroinflammatory ageing mechanisms. Understanding these microbial shifts and their interactions with the gut–brain axis may enable the development of microbiome-based interventions to mitigate neurodegeneration.

Among various strategies to restore microbial balance, probiotics have shown promising effects in counteracting gut dysbiosis and age-associated inflammation [16,17,18]. Probiotics—defined as live microorganisms that confer health benefits when administered in adequate amounts—can help restore microbial composition, reduce chronic inflammation and brain oxidative stress, and improve memory and lifespan, as observed in model organisms [19,20]. For example, Hor et al. [21] demonstrated that administration of *Lactobacillus paracasei* OFS 0291 and *Lactobacillus helveticus* OFS 1515 reduced opportunistic *Bacteroides* species in D-galactose-induced rats, while *Lactobacillus fermentum* DR9 supplementation increased *Lactobacillus* abundance and acetate levels. These findings suggest that probiotics may beneficially reshape gut microbial composition and metabolite production in the ageing gut.

Building on this concept, the present preliminary study explored the potential modulatory effects of Malaysian kefir water (a traditionally fermented beverage rich in *Lactobacillus* and *Bifidobacterium* species) on gut microbiota composition in D-galactose-induced ageing BALB/c mice. The D-galactose (D-gal)-induced ageing model was used as it closely mimics natural ageing processes through chronic oxidative stress and the accumulation of advanced glycation end products, leading to inflammation and neurodegeneration, making it a well-established system for studying age-related physiological and microbial changes, including gut dysbiosis [22].

Kefir water, composed primarily of lactic acid bacteria, has previously been shown to modulate gut microbiota and improve gut barrier function [23,24]. Our previous research demonstrated that kefir water possesses antioxidant and neuroprotective activities, attenuating oxidative stress in human SH-SY5Y neuronal cells [25], while ultra-high-performance liquid chromatography analysis identified flavonoid and phenolic acid derivatives as key bioactive constituents [26]. However, its in vivo effects on gut microbial modulation during ageing remain largely unexplored.

Therefore, this study aimed to explore the feasibility of using kefir water as a dietary intervention to modulate gut microbial composition in an ageing mouse model. Specifically, we evaluated kefir water as a pre-treatment and co-treatment to examine its potential influence on microbial diversity, composition, and *Lactobacillus* enrichment. The findings from this exploratory investigation provide preliminary insight into the possible gut microbiota-modulating capacity of Malaysian kefir water, laying the groundwork for more comprehensive future studies on its anti-ageing and neuroprotective potential.

## 2. Materials and Methods

### 2.1. Preparation of Kefir Water

Kefir water grains were purchased from a local store, Kefir and Kombucha, in Kuala Lumpur, Malaysia (https://www.kefirandkombucha.com, accessed on 1 June 2021). The kefir water was prepared as described in our previous studies [25,26].

### 2.2. Experimental Animals and Ethics Statement

This study received ethical approval from Universiti Putra Malaysia’s Institutional Animal Care and Use Committee (UPM/IACUC/AUP-R014/2020) and was conducted in accordance with their guidelines. All procedures complied with OECD and ARRIVE guidelines. A total of 48 male BALB/c mice (7 weeks old, weighing 18–22 g) were obtained from the Animal House of the Faculty of Veterinary Medicine, Universiti Putra Malaysia.

### 2.3. Experimental Design

Mice were acclimatised for 7 days upon arrival. They were housed in plastic cages at 22 ± 1 °C, with a 12 h light/dark cycle and approximately 60% relative humidity. Mice were provided with a standard pellet diet and distilled water ad libitum throughout the experiment. The dietary composition of the standard pellet (AIN-93G) is provided in Table S1.

**Figure 1:**
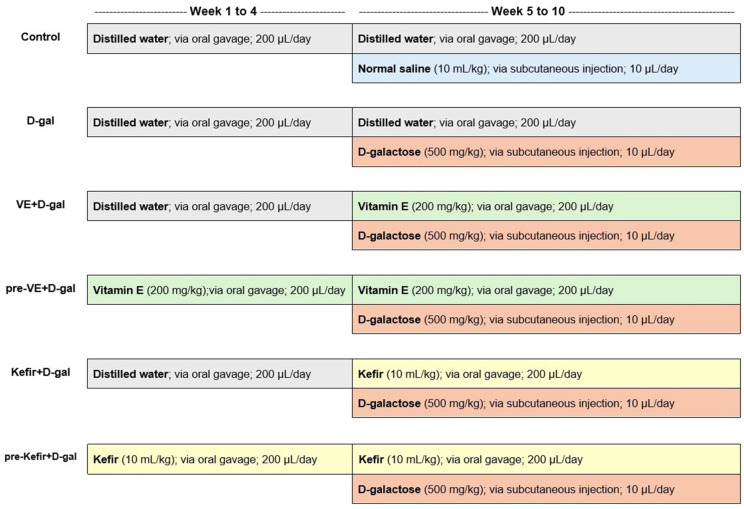
Overview of the experimental design and treatment administration. Mice were acclimatised for 7 days before the experiment commenced in Week 1 and concluded in Week 10. Mice in the pre-VE + D-galactose and pre-Kefir + D-galactose groups received 200 µL of VE (200 mg/kg) and 200 µL of Kefir (10 mL/kg), respectively, for 28 days (Week 1–Week 4) via oral gavage, while mice in the other groups received 200 µL of distilled water. Starting from Week 5, when the mice reached 12 weeks of age, all groups except for the control were administered 10 µL of D-galactose (500 mg/kg) subcutaneously, followed by 200 µL of VE (200 mg/kg) or 200 µL of Kefir (10 mL/kg) via oral gavage until Week 10. The control group received 10 µL of sterile normal saline subcutaneously along with 200 µL of distilled water via oral gavage.

After one week of acclimatisation, mice were randomly assigned to six groups (*n* = 8 per group) and treated daily for ten weeks as follows: (i) the control group served as the normal control, (Control, *n* = 8), (ii) D-gal group served as the negative control (D-gal, *n* = 8), (iii) the vitamin E + D-gal group served as the co-treatment positive control (VE+D-gal, *n* = 8), (iv) the pre-treatment vitamin E + D-gal group served as the pre-treatment positive control (pre-VE + D-gal, *n* = 8), (v) the kefir + D-gal group served as the co-treatment with kefir water (Kefir + D-gal, *n* = 8), and (vi) the pre-treatment kefir + D-gal group served as the pre-treatment with kefir water (pre-Kefir+D-gal, *n* = 8). The experimental design and treatment administration are summarised in Figure 1. Treatments, including kefir water (10 mL/kg/day), VE (200 mg/kg/day), and distilled water, were administered via oral gavage at 200 µL, while D-gal was administered via subcutaneous injection at 10 µL of 500 mg/kg/day to the respective treatment groups.

The concentrations of D-gal and vitamin E, as well as the treatment duration, were selected based on previous studies [27,28]. According to the study timeline (Figure 1), all mice except those in the control group were induced with D-gal at week 5 (when mice were 12 weeks old), an age considered suitable for D-gal induction [27,28,29]. Changes in physical appearance, behaviour, and body weight were recorded weekly. At the end of the treatment period, faecal samples were collected into sterilised 1.5 mL tubes and stored at −80 °C for 16S rRNA metagenomic analysis. Mice were sacrificed via terminal exsanguination under general anaesthesia using ketamine-xylazine (100 mg/kg: 10 mg/kg), administered intraperitoneally.

### 2.4. Faecal DNA Extraction

Genomic DNA was extracted from mouse faeces using the QIAamp^®^ Fast DNA Stool Mini Kit (Qiagen, Hilden, Germany) following the manufacturer’s protocol. Briefly, 220 mg of faeces was homogenised in 1 mL InhibitEX buffer using a D1000-E handheld homogeniser (Benchmark Scientific, Sayreville, NJ, USA) and incubated at 95 °C for 15 min to lyse microbial cells. The sample was centrifuged at 14,000× *g* for 10–15 min, and 200 µL of the supernatant was mixed with 15 µL Proteinase K and 200 µL Buffer AL, followed by incubation at 70 °C for 20 min. An equal volume of ethanol (96–100%) was then added, and the lysate was transferred to a QIAamp spin column for purification according to the manufacturer’s instructions. DNA yield and quality were assessed using a Qubit 2.0 Fluorometer (Life Technologies, Waltham, MA, USA) and an Agilent Bioanalyzer 2100 (Agilent Technologies, Santa Clara, CA, USA). Extracted DNA was stored at −20 °C until further use.

### 2.5. Amplicon Generation

Purified genomic DNA that passed quality control (QC) was used as a template for amplification with locus-specific primers as follows: 

Forward primer: CCTACGGGNGGCWGCAG

Reverse primer: GACTACHVGGGTATCTAATCC

PCR reactions were performed using REDiant 2× PCR Master Mix (1st BASE, Singapore) following the manufacturer’s protocol. A 50 µL reaction contained REDiant 2× PCR Master Mix (1×), forward and reverse primers (1 µM each), 5 ng DNA template, and nuclease-free water. Reactions were briefly centrifuged and amplified in a thermal cycler with an initial denaturation at 95 °C for 5 min, followed by 25–35 cycles of 95 °C for 1 min, 65 °C for 1 min, and 72 °C for 1 min, with a final extension at 72 °C for 15 min.

### 2.6. Quantification and Qualification of PCR Products

The ~450 bp PCR products were electrophoresed on a 1% (*w*/*v*) Tris–acetate EDTA (TAE) agarose gel and visualised under UV light using the Bio-Rad Imager documentation system (Bio-Rad, Hercules, CA, USA).

### 2.7. Primary Phase of Library Construction (1st Stage PCR)

The 16S rRNA gene amplicons were prepared for sequencing on the Illumina MiSeq System (Illumina, San Diego, CA, USA) using the two-stage PCR protocol described in the Illumina 16S Metagenomic Library Preparation Guide. In the first PCR stage, the bacterial 16S rRNA gene targeting the V3–V4 regions was amplified using locus-specific primers with Illumina overhang adapters as follows:

Forward overhang:

27F 5′-TCGTCGGCAGCGTCAGATGTGTATAAGAGACAG-[CCTACGGGNGGCWGCAG]-3′

Reverse overhang:

1492R 5′-GTCTCGTGGGCTCGGAGATGTGTATAAGAGACAG-[GACTACHVGGGTATCTAATCC]-3′

PCR reactions were performed using KOD-Multi & Epi^®^ (Toyobo, Osaka, Japan) according to the manufacturer’s protocol. Each 50 µL reaction contained 2× PCR buffer (1×), forward and reverse primers (0.3 µM each), ≤50 ng DNA template, 1 µL KOD-Multi & Epi^®^, and PCR-grade water. Reactions were briefly centrifuged and amplified in a thermal cycler with an initial denaturation at 94 °C for 2 min, followed by 30–40 cycles of 98 °C for 10 s, 65 °C for 10 s, and 68 °C for 30 s.

### 2.8. Secondary Phase of Library Construction (2nd Stage PCR)

Dual indices were attached to the amplicon PCR using the Illumina Nextera XT Index Kit v2 according to the manufacturer’s protocol (Agilent Technologies, Santa Clara, CA, USA). Briefly, 5 µL of PCR product was combined with Nextera XT Index Primers 1 (N7xx) and 2 (S5xx) (5 µL each), 2× KAPA HiFi HotStart ReadyMix (25 µL), and PCR-grade water (10 µL) in a 50 µL reaction. The mixture was gently mixed, covered with Microseal ‘A’, and briefly centrifuged. PCR amplification consisted of an initial denaturation at 95 °C for 3 min, followed by 8 cycles of 95 °C for 30 s, 55 °C for 30 s, and 72 °C for 30 s, with a final extension at 72 °C for 5 min. Library quality was assessed using the Agilent Bioanalyzer 2100 System with the Agilent DNA 1000 Kit, and fluorometric quantification was performed using Helixyte Green^TM^ (AAT Bioquest^®^, Inc., Pleasanton, CA, USA).

### 2.9. Next-Generation Sequencing

Libraries were normalised and pooled according to Illumina’s recommended protocol and sequenced on the MiSeq platform (Illumina, San Diego, CA, USA) using a 300-cycle paired-end (PE) run. The final concentrated library was diluted to 4 nM with resuspension buffer (RSB) or 10 mM Tris (pH 8.5), and 5 µL from each diluted library was pooled to create the final sequencing mix containing unique index combinations.

### 2.10. Metagenomics Data Analysis

Raw sequencing data from Illumina 16S rRNA metagenomics sequencing were processed using the QIIME2 v.2020.06 pipeline (https://doi.org/10.1038/s41587-019-0209-9, accessed on 28 July 2022). Sequence adaptors and low-quality reads were trimmed using BBDuk from the BBTools package (https://sourceforge.net/projects/bbmap/, accessed on 28 July 2022), and paired-end reads were merged with USEARCH v11.0.667 (https://www.drive5.com/usearch/, accessed on 28 July 2022). After filtering out reads shorter than 150 bp or longer than 600 bp, error correction and chimera removal were performed using the DADA2 v1.18 pipeline (https://benjjneb.github.io/dada2/, accessed on 28 July 2022). The resulting amplicon sequence variants (ASVs) were aligned against the SILVA 132 16S rRNA database using VSEARCH v2.6.2. Operational taxonomic units (OTUs) were clustered at 97% similarity with UPARSE v11.0.667, and low-abundance OTUs (fewer than two reads) were excluded from downstream analysis. Multiple sequence alignments were generated using MAFFT, and a phylogenetic tree for UniFrac analysis was constructed with FastTree2.

### 2.11. Statistical Analysis

Data analysis was performed using R Studio (version 3.6.2) with the phyloseq package (https://www.bioconductor.org/packages/release/bioc/html/phyloseq.html, accessed on 28 July 2022) for metagenomic bioinformatics analysis. The Kruskal–Wallis test, a non-parametric method suitable for small sample sizes and non-normally distributed data, was performed to compare treatment groups. However, no significant differences were detected, which may be attributed to the limited number of biological replicates. Observed outcomes were therefore interpreted descriptively to identify potential trends in gut microbiota modulation. As this is a preliminary study, future investigations will include 5–10 biological replicates per group to ensure adequate statistical power and allow for more robust functional validation.

## 3. Results

### 3.1. Effects of Kefir Water on the Alpha Diversity of Gut Microbial Species

A total of 1,761,238 sequence reads were obtained from 12 faecal samples (2 per group). After trimming and quality filtering, 84.75% of reads were retained, resulting in 1,492,634 high-quality sequences, with an average of 56,725 per sample (Table S2).

Alpha diversity, assessed through observed operational taxonomic units (OTUs), species richness (Abundance-based coverage estimator (ACE)), and diversity indices (Chao1, Shannon, Simpson), is summarised in Table 1. Operational taxonomic units which represent clusters of microbial taxa based on sequence similarity, are commonly used to assess microbial richness and community composition. Abundance-based coverage estimator and Chao1 estimate species richness, accounting for both abundant and rare taxa, while Shannon and Simpson indices consider both richness and evenness to describe overall diversity.

Both kefir water-treated groups showed comparatively lower observed OTUs (Kefir + D-gal: 281.00 ± 22.00; pre-Kefir + D-gal: 308.50 ± 4.50) relative to the D-gal (338.50 ± 0.50), control (359.50 ± 5.50), VE co-treated (316.00 ± 33.00), and VE pre-treated (433.00 ± 2.00) groups (*p* = 0.097). Similarly, kefir-treated mice exhibited lower species richness (ACE: 281.10 ± 22.10 and 311.87 ± 5.68; *p* = 0.097) and diversity indices (Chao1: 281.00 ± 22.00 and 315.17 ± 8.83 (*p* = 0.096); Shannon: 4.73 ± 0.12 and 4.48 ± 0.07 (*p* = 0.095); Simpson: 57.19 ± 10.52 and 46.33 ± 4.09 (*p* = 0.122)) compared to other groups. In contrast, the pre-VE+D-gal group had the highest OTUs (433.00 ± 2.00, *p* = 0.097), species richness (ACE: 435.28 ± 2.66; *p* = 0.097), and diversity indices (Chao1: 435.11 ± 2.44 (*p* = 0.096); Shannon: 5.09 ± 0.08 (*p* = 0.095); Simpson: 82.45 ± 10.45 (*p* = 0.122)).

### 3.2. Effects of Kefir Water on UPGMA Tree Analysis

**Figure 2:**
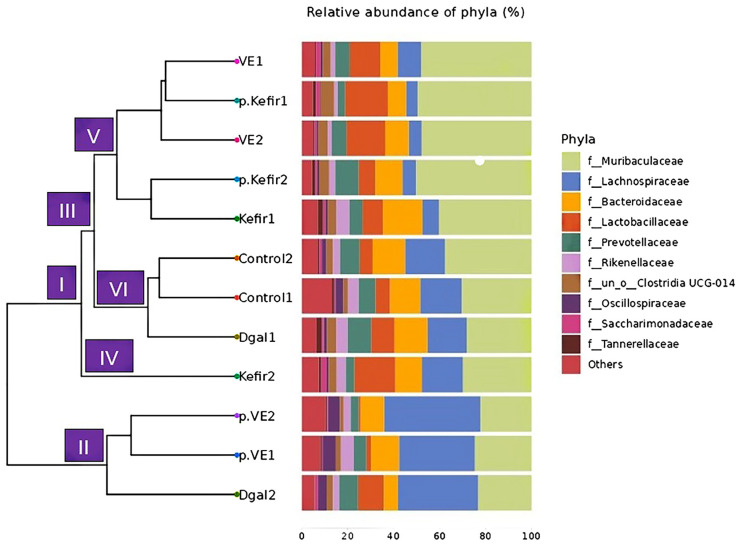
UPGMA tree and relative abundance of bacterial phyla. Left: UPGMA clustering tree showing phylogenetic relationships among samples. Right: Horizontal bar chart representing the percentage of relative abundance of bacterial phyla. Shorter branch lengths in the UPGMA tree indicate greater similarity in phylum-level composition between samples, while longer branch lengths represent higher dissimilarity. Control: normal mice without induced ageing; D-gal: negative control mice administered 500 mg/kg/day D-gal; Kefir: kefir water co-treated mice administered 10 mL/kg/day kefir water and 500 mg/kg/day D-gal; p.Kefir: kefir water pre-treated mice pre-administered 10 mL/kg/day kefir water before receiving 500 mg/kg/day D-gal; VE: vitamin E co-treated mice administered 200 mg/kg/day VE and 500 mg/kg/day D-gal; p.VE: vitamin E pre-treated mice pre-administered 200 mg/kg/day VE before receiving 500 mg/kg/day D-gal.

Beta diversity, assessed via hierarchical clustering using the unweighted pair group method with arithmetic mean (UPGMA) tree, was applied to evaluate compositional differences among treatment groups. The UPGMA analysis classified the microbial communities into two main clusters, designated as Groups I and II, both originating from a common ancestral root (Figure 2). Group II primarily consisted of the pre-VE+D-gal treatment, whereas Group I encompassed the control, D-gal (sample 1), kefir-treated, and VE-co-treated groups.

Within Group I, additional branching was observed, forming two sub-clusters (III and IV), with sub-cluster III further dividing into branches V and VI. The phylogenetic distribution revealed that the control and D-gal (sample 1) (branch VI) clustered closely, while the kefir pre-treated, kefir co-treated, and VE-co-treated groups (branches V and IV) diverged into distinct yet related lineages. Interestingly, D-gal (sample 2) grouped with the pre-VE+D-gal treatment (Group II) rather than clustering with D-gal (sample 1), suggesting a degree of intra-group variability among the D-gal samples.

### 3.3. Beta-Diversity Analysis Based on KEGG Orthologues

**Figure 3:**
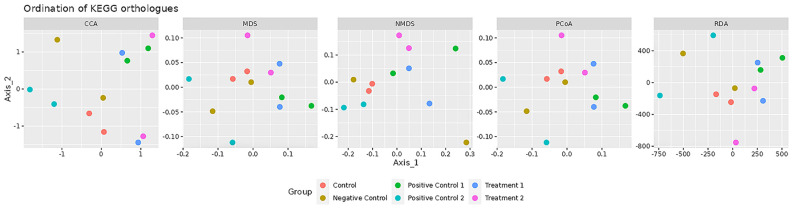
Beta-diversity analysis of gut microbial functional profiles based on KEGG orthologue abundances. Ordination methods include Canonical Correspondence Analysis (CCA), Redundancy Analysis (RDA), Multidimensional Scaling (MDS), Non-metric Multidimensional Scaling (NMDS), and Principal Coordinates Analysis (PCoA). Control (red): normal mice without induced ageing; D-gal (Negative Control, mustard): mice administered 500 mg/kg/day D-gal; Kefir (Treatment 1, blue): kefir water co-treated mice administered 10 mL/kg/day kefir water and 500 mg/kg/day D-gal; p.Kefir (Treatment 2, purple): kefir water pre-treated mice pre-administered 10 mL/kg/day kefir water before receiving 500 mg/kg/day D-gal; VE (Positive Control 1, green): vitamin E co-treated mice administered 200 mg/kg/day VE and 500 mg/kg/day D-gal; p.VE (Positive Control 2, cyan): vitamin E pre-treated mice pre-administered 200 mg/kg/day VE before receiving 500 mg/kg/day D-gal.

To examine functional differences in the gut microbiome across treatment groups, multiple ordination analyses were performed using KEGG orthologue profiles, including Canonical Correspondence Analysis (CCA), Redundancy Analysis (RDA), Multidimensional Scaling (MDS), Non-metric Multidimensional Scaling (NMDS), and Principal Coordinates Analysis (PCoA) (Figure 3).

CCA and RDA, as constrained ordination methods, assess how well treatment groups explain variation in microbial functional profiles. In both analyses, kefir co-treated (Treatment 1, blue) and pre-treated (Treatment 2, purple) groups formed clusters distinct from the control (red), D-gal (Negative Control, mustard), and VE pre-treatment (Positive Control 2, cyan) groups. Notably, the kefir groups were positioned closer to the VE co-treatment group (Positive Control 1, green), indicating that kefir administration induced functional shifts in the gut microbiome that were more similar to VE co-treatment than to the untreated control.

MDS, NMDS, and PCoA, as unconstrained ordination methods, visualised overall sample similarity without prior group constraints. These plots show a comparable pattern: the control and D-gal groups clustered separately, while the kefir-treated groups consistently appeared near the VE co-treatment cluster. Partial overlap between kefir and VE co-treatment was observed, suggesting that kefir treatments shared functional features with VE co-treatment.

Overall, these ordinations indicate that kefir water pre-treatment and co-treatment modulate the gut microbiome in a manner more aligned with VE co-treatment than with the untreated control. This preliminary observation suggests that kefir and VE co-treatment may exert comparable modulatory effects on microbial functional profiles. However, further targeted analyses with larger sample sizes are required to identify the specific functional pathways involved.

### 3.4. Effects of Kefir Water on the Rank Abundance and Rarefaction Curves of Gut Microbiota

The rank abundance and rarefaction curves (Figures S1 and S2) were used to provide an overview of microbial diversity and sequencing depth across the treatment groups. The rank abundance curves revealed that kefir pre-treatment exhibited the widest and smoothest profile, suggesting greater species richness and more even distribution of taxa. In contrast, the VE co-treatment group displayed a steep decline, indicative of a few dominant species and lower evenness, while the other groups showed intermediate diversity patterns.

Similarly, the rarefaction curves demonstrated variation in observed species richness among the groups. The VE pre-treatment group exhibited the highest richness, whereas kefir water pre- and co-treatment groups reached saturation at lower read depths, suggesting a moderate microbial diversity compared to controls. These findings, though preliminary, provide supportive evidence of treatment-dependent modulation in microbial richness and evenness.

### 3.5. Effects of Kefir Water on the Phylum-Level Bacterial Taxonomic Composition

**Figure 4:**
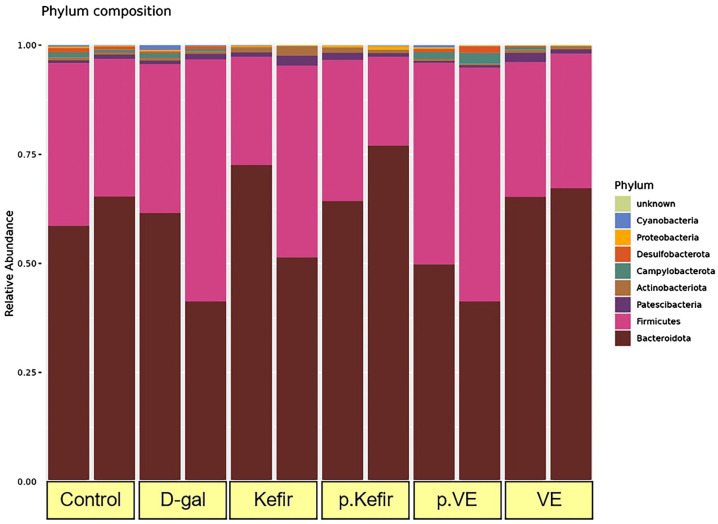
Relative abundance profiles of bacterial taxa at the phylum level across all treatment groups. Results are presented as *n* = 2 per group. Control: normal mice without induced ageing; D-gal: negative control mice administered 500 mg/kg/day D-gal; Kefir: kefir water co-treated mice administered 10 mL/kg/day kefir water and 500 mg/kg/day D-gal; p.Kefir: kefir water pre-treated mice pre-administered 10 mL/kg/day kefir water before receiving 500 mg/kg/day D-gal; VE: vitamin E co-treated mice administered 200 mg/kg/day VE and 500 mg/kg/day D-gal; p.VE: vitamin E pre-treated mice pre-administered 200 mg/kg/day VE before receiving 500 mg/kg/day D-gal.

As shown in Figure 4, eight bacterial phyla were identified in the gut microbiota of D-gal-induced mice across all treatment groups: *Bacteroidota*, *Firmicutes*, *Patescibacteria*, *Actinobacteriota*, *Campylobacterota*, *Desulfobacterota*, *Proteobacteria*, and *Cyanobacteria*. Among these, *Bacteroidota* and *Firmicutes* were the most predominant phyla, though their relative abundances did not differ significantly among groups (*Bacteroidota*: *p* = 0.251; *Firmicutes*: *p* = 0.238, Table 2).

The relative abundance of *Bacteroidota* decreased in the D-gal-treated group (0.515 ± 0.101%) compared to the control group (0.620 ± 0.034%). Interestingly, kefir pre-treatment increased *Bacteroidota* abundance to 0.707 ± 0.064%, while kefir co-treatment maintained a level similar to that of the control (0.620 ± 0.034%). The VE pre-treated group showed the lowest *Bacteroidota* abundance (0.456 ± 0.043%), whereas the VE co-treated group demonstrated a relatively high abundance (0.663 ± 0.010%).

In contrast, *Firmicutes* abundance was elevated in the D-gal-treated group (0.448 ± 0.107%) compared to the control (0.344 ± 0.029%). The kefir pre-treated group showed the lowest *Firmicutes* abundance (0.263 ± 0.060%), while the kefir co-treated group (0.344 ± 0.029%) remained comparable to the control. The VE pre-treated group exhibited the highest *Firmicutes* abundance (0.499 ± 0.037%), whereas the VE co-treated group showed 0.309 ± 0.309%, which was not higher than that of the control or D-gal-treated groups.

### 3.6. Effects of Kefir Water on Bacterial Taxonomic Composition at the Family Level

**Figure 5:**
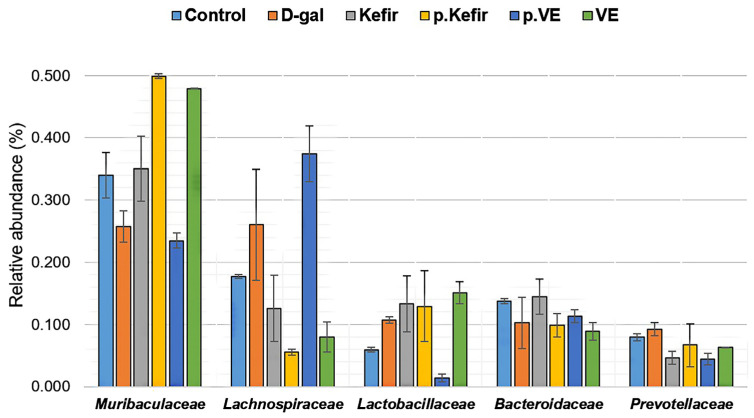
Relative abundance of bacterial taxa at the family level across all treated groups. Data are expressed as mean ± standard deviation (SD). All values showed *p* > 0.05 based on the Kruskal–Wallis test, indicating no significant differences among the groups. The calculated relative abundance values for each family are provided in Table S3. Control: normal mice without induced ageing; D-gal: negative control mice administered 500 mg/kg/day D-gal; Kefir: kefir water co-treated mice administered 10 mL/kg/day kefir water and 500 mg/kg/day D-gal; p.Kefir: kefir water pre-treated mice pre-administered 10 mL/kg/day kefir water before receiving 500 mg/kg/day D-gal; VE: vitamin E co-treated mice administered 200 mg/kg/day VE and 500 mg/kg/day D-gal; p.VE: vitamin E pre-treated mice pre-administered 200 mg/kg/day VE before receiving 500 mg/kg/day D-gal.

A total of 35 bacterial families were identified across all treatment groups (Figure S3). The most notable gut microbial families observed in the kefir water-treated groups—*Muribaculaceae*, *Lachnospiraceae*, *Lactobacillaceae*, *Bacteroidaceae*, and *Prevotellaceae*—are illustrated in Figure 5.

Both kefir water pre-treatment and co-treatment appeared to non-significantly increase the relative abundance of *Muribaculaceae* (pre-Kefir+D-gal: 0.499 ± 0.004; Kefir+D-gal: 0.350 ± 0.052) and *Lactobacillaceae* (pre-Kefir+D-gal: 0.129 ± 0.057; Kefir+D-gal: 0.133 ± 0.045), compared to the D-gal-treated group (0.257 ± 0.025, *p* = 0.069 and 0.107 ± 0.005, *p* = 0.156, respectively). Similarly, kefir water co-treatment resulted in a slight, non-significant increase in *Bacteroidaceae* abundance (0.144 ± 0.028), whereas pre-treatment showed a non-significant decrease (0.099 ± 0.019) compared to the D-gal-treated group (0.103 ± 0.041, *p* = 0.465).

In contrast, the relative abundance of *Lachnospiraceae* and *Prevotellaceae* was non-significantly reduced in both kefir-treated groups (pre-Kefir+D-gal: 0.055 ± 0.004; 0.067 ± 0.034; Kefir+D-gal: 0.126 ± 0.053; 0.047 ± 0.010) compared to the D-gal-treated group (*Lachnospiraceae*: 0.260 ± 0.090, *p* = 0.125; *Prevotellaceae*: 0.092 ± 0.011, *p* = 0.277).

Among the VE-treated groups, an increased abundance of *Muribaculaceae* was observed (0.479 ± 0.001, *p* = 0.069) for the co-treatment group, whereas pre-treatment showed a reduced abundance of *Muribaculaceae* (0.235 ± 0.012, *p* = 0.069) and an increasing abundance of *Lachnospiraceae* (0.374 ± 0.045, *p* = 0.125), relative to the D-gal-treated group (*Muribaculaceae*: 0.257 ± 0.025; *Lachnospiraceae*: 0.260 ± 0.090).

VE pre-treatment also resulted in a lower *Lactobacillaceae* abundance (0.014 ± 0.006, *p* = 0.156), while VE co-treatment showed a higher abundance (0.151 ± 0.018, *p* = 0.156) than the D-gal-treated group (0.107 ± 0.005).

### 3.7. Effects of Kefir Water on Bacterial Taxonomic Composition at the Genus Level

**Figure 6:**
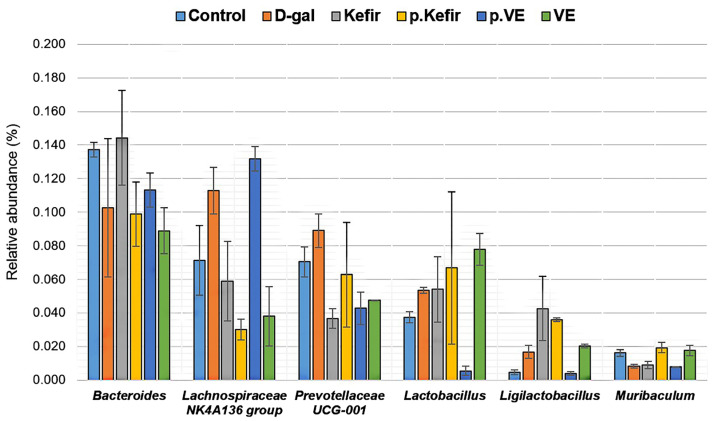
Relative abundance of bacterial taxa at the genus level across all treated groups. Data are expressed as mean ± standard deviation (SD). All values showed *p* > 0.05 based on the Kruskal–Wallis test, indicating no significant differences among the groups. The calculated relative abundance values for each genus are provided in Table S4. Control: normal mice without induced ageing; D-gal: negative control mice administered 500 mg/kg/day D-gal; Kefir: kefir water co-treated mice administered 10 mL/kg/day kefir water and 500 mg/kg/day D-gal; p.Kefir: kefir water pre-treated mice pre-administered 10 mL/kg/day kefir water before receiving 500 mg/kg/day D-gal; VE: vitamin E co-treated mice administered 200 mg/kg/day VE and 500 mg/kg/day D-gal; p.VE: vitamin E pre-treated mice pre-administered 200 mg/kg/day VE before receiving 500 mg/kg/day D-gal.

A total of 71 bacterial genera were identified across all treatment groups, with a relatively high abundance of an unclassified genus observed (Figure S4). At the genus level, notable variations were detected in *Bacteroides*, *Lachnospiraceae* NK4A136 group, *Prevotellaceae* UCG-001, *Lactobacillus*, *Ligilactobacillus*, and *Muribaculum* (Figure 6). However, these changes were not statistically significant (*p* > 0.05).

Kefir water pre-treatment showed a slightly lower relative abundance of *Bacteroides* (0.099 ± 0.019, *p* = 0.465) but an increasing abundance of *Lactobacillus* (0.067 ± 0.045, *p* = 0.257), *Ligilactobacillus* (0.036 ± 0.001, *p* = 0.075), and *Muribaculum* (0.019 ± 0.003, *p* = 0.125), compared to the D-gal-treated group (*Bacteroides*: 0.103 ± 0.041; *Lactobacillus*: 0.054 ± 0.002; *Ligilactobacillus*: 0.017 ± 0.004; *Muribaculum*: 0.008 ± 0.001). Kefir co-treatment similarly showed an increased abundance of *Bacteroides* (0.144 ± 0.028, *p* = 0.465), *Ligilactobacillus* (0.042 ± 0.019, *p* = 0.075), and *Muribaculum* (0.009 ± 0.002, *p* = 0.125), while maintaining comparable *Lactobacillus* abundance (0.054 ± 0.020, *p* = 0.257) relative to the D-gal-treated group.

Both kefir water treatments, however, showed a trend toward reduced abundance of *Lachnospiraceae* NK4A136 group (Kefir+D-gal: 0.059 ± 0.023; pre-Kefir+D-gal: 0.030 ± 0.006) and *Prevotellaceae* UCG-001 (Kefir+D-gal: 0.037 ± 0.006; pre-Kefir+D-gal: 0.063 ± 0.031) compared to the D-gal-treated group (*Lachnospiraceae* NK4A136: 0.113 ± 0.014, *p* = 0.125; *Prevotellaceae* UCG-001: 0.089 ± 0.010, *p* = 0.271).

Among the VE-treated groups, pre-treatment demonstrated an increased *Lachnospiraceae* NK4A136 group (0.132 ± 0.007, *p* = 0.125) but reduced *Ligilactobacillus* (0.004 ± 0.001, *p* = 0.075) and *Lactobacillus* (0.006 ± 0.003, *p* = 0.257) compared to the D-gal-treated group. In contrast, VE co-treatment showed slightly increased *Ligilactobacillus* (0.020 ± 0.001, *p* = 0.075) and *Lactobacillus* (0.078 ± 0.009, *p* = 0.257), while showing a reduced abundance of *Prevotellaceae* UCG-001 (0.047 ± 0.000, *p* = 0.271). The abundance of *Muribaculum* remained comparable to kefir-treated groups, with an observable increase in the VE co-treatment group (0.018 ± 0.003, *p* = 0.125) and unchanged levels in the VE pre-treatment group (0.008 ± 0.001, *p* = 0.125).

### 3.8. Effect of Kefir Water on the Abundance of Lactobacillus, Ligilactobacillus, and HT002

**Figure 7:**
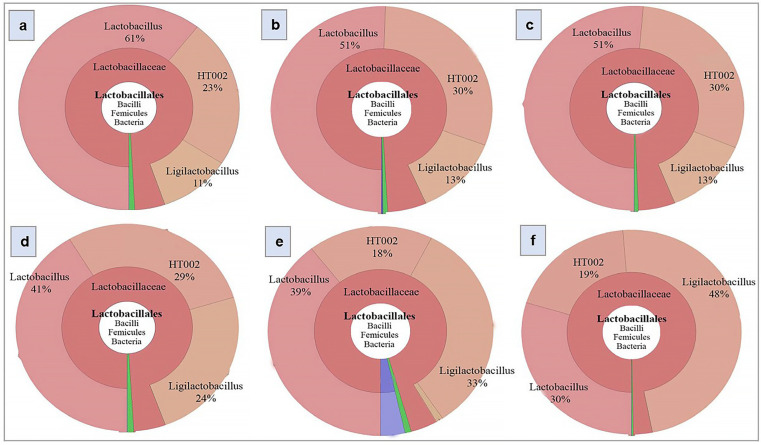
Krona chart illustrating the relative abundance of the bacterial genera *Lactobacillus*, *Ligilactobacillus*, and HT002 across treatment groups: (**a**) Control, (**b**) D-gal, (**c**) VE+D-gal, (**d**) pre-VE+D-gal, (**e**) Kefir+D-gal, and (**f**) pre-Kefir+D-gal. Control: normal mice without induced ageing; D-gal: negative control mice administered 500 mg/kg/day D-gal; Kefir: kefir water co-treated mice administered 10 mL/kg/day kefir water and 500 mg/kg/day D-gal; p.Kefir: kefir water pre-treated mice pre-administered 10 mL/kg/day kefir water before receiving 500 mg/kg/day D-gal; VE: vitamin E co-treated mice administered 200 mg/kg/day VE and 500 mg/kg/day D-gal; p.VE: vitamin E pre-treated mice pre-administered 200 mg/kg/day VE before receiving 500 mg/kg/day D-gal. Note: The Krona visualisation represents the microbial composition from a representative biological replicate within each treatment group.

A Krona chart was used to visualise the relative abundance of bacterial taxa across multiple phylogenetic levels within each sample (Figure 7). Notably, the distribution of *Lactobacillus* and *Ligilactobacillus* varied among treatment groups. As shown in Table 3, the control group was dominated by *Lactobacillus* (63%), followed by *Ligilactobacillus* (8%). In D-gal-treated mice, the relative abundance of *Lactobacillus* decreased to 50% (*p* = 0.202), accompanied by a moderate increase in *Ligilactobacillus* (15.5%, *p* = 0.098). A comparable trend was observed in the VE co-treatment group, with *Lactobacillus* and *Ligilactobacillus* comprising 51% (*p* = 0.202) and 13.5% (*p* = 0.098) of the microbiota, respectively.

In contrast, VE pre-treatment led to a reduction in *Lactobacillus* abundance (38%, *p* = 0.202) and a corresponding increase in *Ligilactobacillus* (30%, *p* = 0.098) relative to the D-gal group. Kefir water treatments induced more pronounced and balanced shifts in these genera, particularly favouring *Ligilactobacillus*. In the kefir co-treatment group, *Lactobacillus* decreased to 38.5% (*p* = 0.202), while *Ligilactobacillus* increased to 29.5% (*p* = 0.098). Similarly, kefir pre-treatment showed in 45% *Lactobacillus* (*p* = 0.202) and 34% *Ligilactobacillus* (*p* = 0.098).

Additionally, a taxon identified as HT002, a bacterium within the *Lactobacillus* genus of the *Lactobacillaceae* family, was consistently detected across all treatment groups (Figure 6, Table 3). Its relative abundance was 24% in the control group, increasing to 28.5% in both the D-gal-treated and VE pre-treated groups (*p* = 0.149). A slight increase was observed in the VE co-treatment group (29.5%, *p* = 0.149), whereas kefir water co- and pre-treatments showed lower levels (23% and 17.5%, *p* = 0.149, respectively).

## 4. Discussion

The 16S rRNA metagenomic analysis of alpha and beta diversity revealed that kefir water pre-treatment and co-treatment did not produce statistically significant changes in gut microbial species richness or diversity under the conditions tested. Nevertheless, several notable trends were observed. The D-gal-treated group showed a reduction in alpha diversity compared to the control group, consistent with previous findings linking ageing and oxidative stress to decreased microbial richness [21,22]. Interestingly, both kefir water-treated groups demonstrated a further decline in alpha diversity, a pattern also observed in the vitamin E co-treated group. Although this reduction could suggest a lower microbial diversity, the rarefaction and rank abundance curves provide additional insight. The kefir-treated groups showed lower rarefaction profiles but higher rank abundance dominance, indicating that kefir water may promote the selective modulation of specific microbial taxa while maintaining a lower overall diversity. In particular, the combination of lower alpha diversity and a high rank-abundance curve suggests a selective enrichment pattern, where a few dominant species are favoured over broader community diversity. This pattern may reflect an early stage of microbial adaptation, where selective taxa expand in abundance before a broader diversification occurs. In contrast, the vitamin E pre-treated group displayed higher OTUs, ACE, and alpha diversity indices than the other groups, suggesting that early antioxidant intervention may help stabilise microbial richness under ageing-associated stress. Collectively, these observations suggest that kefir water may influence gut microbial ecology through targeted compositional shifts rather than broad diversity enhancement, reflecting an adaptive modulation within the ageing gut microenvironment.

These findings are consistent with previous reports showing that kefir modulates gut microbiota composition more than overall diversity. For instance, Desnilasari et al. [30] observed that kefir and kefir–glucomannan supplementation in rats with metabolic syndrome did not significantly change microbial diversity but selectively enriched *Lactobacillus* (14.61%) and *Bifidobacterium* (2.2%) while reducing *Clostridium* (38.15%) and *Bacteroides* (22.51%). Similarly, Bellikci-Koyu et al. [23] reported enrichment of *Actinobacteria* following kefir consumption without significant alterations in other dominant phyla such as *Bacteroidetes*, *Proteobacteria*, and *Verrucomicrobia*.

Consistent with these studies, Bourrie et al. [31] also found that kefir supplementation in high-fat diet-fed mice did not significantly alter microbial community structure or increase alpha diversity, while Wastyk et al. [32] reported no increase in alpha diversity following fermented food consumption in humans. Gupta et al. [24] similarly observed no increase in alpha diversity among critically ill patients, although gut microbiome wellness indices improved. Together, these findings suggest that kefir’s beneficial effects may not stem from enhancing overall microbial diversity but from the selective enrichment of specific beneficial taxa and their metabolites. Descriptively, this study suggests that kefir water promotes adaptive microbial shifts that could precede a broader ecological rebalancing, warranting further investigation with larger cohorts and extended treatment durations.

The dominant bacterial taxa at the phylum, family, and genus levels across the treatment groups are shown in Figure 4, Figure 5 and Figure 6. At the phylum level, *Bacteroidota* and *Firmicutes* were the most abundant. In the D-gal-treated group, there was an increase in *Firmicutes* and a decrease in *Bacteroidota* which led to an elevated *Firmicutes*/*Bacteroidota* ratio, a pattern often associated with gut dysbiosis [33,34]. This observation aligns with previous reports where D-gal administration in mice was linked to an increased *Firmicutes*/*Bacteroidota* ratio, indicative of gut dysbiosis [33,34]. Notably, kefir water co-treatment appeared to reverse this trend, restoring the *Firmicutes*/*Bacteroidota* ratio to levels similar to the control group, suggesting a potential rebalancing of the gut microbial profile. Kefir pre-treatment for 28 days further reduced the *Firmicutes*/*Bacteroidota* ratio due to increased *Bacteroidota* and decreased *Firmicutes* abundance. This observation is consistent with findings by Peluzio et al. [35], who reported that kefir administration reduced *Firmicutes* and *Proteobacteria* while increasing *Bacteroidetes*, *Lactobacillus*, and *Lactococcus*. Similar effects were also noted by Kim et al. [36] and Bellikci-Koyu et al. [23], where kefir supplementation modulated the gut microbiota, enhancing *Bacteroidota* abundance and reducing *Firmicutes*. These trends suggest that kefir supplementation may promote a favourable shift in the gut microbial composition, increasing *Bacteroidota* while reducing *Firmicutes*, potentially due to the probiotic content and the fermentation-derived metabolites present in kefir, which could create a gut environment supportive of certain beneficial bacterial taxa, particularly those classified under *Bacteroidota* [23,35,36].

The dominant bacterial families observed in this preliminary study were *Bacteroidaceae*, *Muribaculaceae*, and *Prevotellaceae* within the phylum *Bacteroidota*, and *Lachnospiraceae* and *Lactobacillaceae* within *Firmicutes* (Figure 4). The predominant genera included *Bacteroides*, *Lachnospiraceae* NK4A136 group, *Prevotellaceae* UCG-001, *Lactobacillus*, *Ligilactobacillus*, and *Muribaculum*. D-galactose treatment reduced the abundance of *Muribaculaceae* (and its genus *Muribaculum*) and *Bacteroidaceae* (and its genus *Bacteroides*), while increasing *Lachnospiraceae* (and its genus *Lachnospiraceae* NK4A136 group) and *Prevotellaceae* (and its genus *Prevotellaceae* UCG-001). These compositional changes align with previous studies reporting similar bacterial shifts associated with ageing and gut dysbiosis [37,38].

Conversely, both kefir water pre-treatment and co-treatment appeared to modulate the gut microbiota in an opposing manner to D-gal treatment. Kefir administration increased the abundance of *Muribaculaceae* (*Muribaculum*) and *Lactobacillaceae* (and its genera *Lactobacillus* and *Ligilactobacillus*), while reducing *Lachnospiraceae* (*Lachnospiraceae* NK4A136 group) and *Prevotellaceae* (*Prevotellaceae* UCG-001). These compositional trends suggest that kefir water may alleviate ageing-associated dysbiosis by selectively enriching potentially beneficial bacterial taxa, even though the changes were not statistically significant.

Interestingly, the D-gal-treated group also showed a higher relative abundance of *Lactobacillaceae* and *Lactobacillus* compared to the control group. A plausible explanation is that certain *Lactobacillus* species are capable of surviving and transiently proliferating under stressful conditions, such as oxidative stress, potentially as an adaptive or compensatory mechanism to maintain gut homeostasis. Previous studies have reported similar transient proliferation of *Lactobacillus* strains under stress or inflammatory conditions [39,40]. However, this selective increase does not necessarily reflect a healthier microbial state, as it occurs alongside other dysbiotic features, such as the increased *Firmicutes*/*Bacteroidota* ratio observed in the D-gal group. These findings represent preliminary observations and warrant further validation with larger sample sizes and functional analyses to confirm the modulatory effects of kefir water on gut microbiota during ageing.

*Muribaculaceae* has recently attracted attention as a promising next-generation probiotic due to its role in host health. It primarily produces short-chain fatty acids (SCFAs), especially propionate and interacts with other probiotics such as *Bifidobacterium* and *Lactobacillus* to support SCFA production [41,42]. Propionate is associated with enhanced gut barrier function, immune modulation, and lifespan extension in mice [42]. Similar observations were reported by Albuquerque et al. [43], where kefir administration increased *Muribaculaceae* abundance in Salmonella Typhimurium-infected C57BL/6 mice.

Kefir is naturally rich in *Lactobacillaceae*, and several studies have reported that its consumption can increase the abundance of *Lactobacillus* and *Ligilactobacillus* in the host gut, as observed in the present study [24,30,32,36]. This is thought to be due to kefir’s polysaccharide matrix, which harbours a microbiota dominated by *Lactobacillus* species. The formation of kefir grains begins with the self-aggregation of *Lactobacillus kefiranofaciens* and *Saccharomyces* spp., forming small granules. Subsequently, *Lentilactobacillus kefiri*, a known biofilm producer, adheres to the surface of these granules and co-aggregates with other microorganisms and milk- or water-derived components to form mature kefir grains [44]. As a result, the fermented kefir solution, whether derived from milk or water, typically contains a microbiota enriched in *Lactobacillus* species, consistent with our previous findings where 16S rRNA sequencing of kefir water revealed *Lactobacillus hilgardii*, *Lactobacillus harbinensis*, *Lactobacillus satsumensis*, and *Lactobacillus zeae* as the most abundant species [26]. These species were also associated with higher levels of flavonoids and phenolic acids, as key bioactive compounds contributing to the antioxidant and neuroprotective effects of kefir water [25].

Kefir-derived *Lactobacillus* has additional functional roles. Van et al. [45] reported that kefir increased the gut microbial capacity to produce γ-aminobutyric acid (GABA) via enrichment of *Lactobacillus reuteri*, which was linked to improving memory and reducing repetitive behaviours in mice. *Lactobacillus* species, as dominant lactic acid bacteria in the human diet, contribute to SCFA production (acetate, propionate, butyrate), which exerts anti-inflammatory effects by modulating NF-κB signalling, inactivating regulatory T-cells, and reducing pro-inflammatory cytokine production [46]. They also strengthen the intestinal barrier, preserving immune tolerance and mitigating gut-related disorders such as irritable bowel syndrome, gastrointestinal infections, and inflammatory bowel disease. Moreover, *Lactobacillus* converts glutamate into GABA, a CNS neurotransmitter that inhibits neuronal excitability and may alleviate pain [47]. Zhang et al. [48] further demonstrated that fermented *Lactobacillus kefiri* supernatant modulated autophagy, oxidative stress, and ageing-related gene expression in hydrogen peroxide-damaged human skin fibroblasts, delaying cellular ageing and senescence.

The Krona results showed that both kefir water co-treatment and pre-treatment appeared to enrich the intestines of D-gal-induced mice with *Ligilactobacillus* compared to the D-gal-treated and control groups. A noticeable shift was observed in the relative abundance from *Lactobacillus* to *Ligilactobacillus*. While the D-gal-treated and control groups showed relative abundances of 15.5% and 8%, respectively, the kefir co-treatment and pre-treatment groups exhibited higher abundances of 29.5% and 34%, suggesting that either early or concurrent kefir administration may promote more stable colonisation of *Ligilactobacillus* in the gut. Although these changes were not statistically significant, the trends suggest that kefir water may favour the enrichment of *Ligilactobacillus* over *Lactobacillus* under ageing-associated conditions. This pattern could indicate a selective modulatory effect, where kefir water supports the growth of specific beneficial taxa linked to gut microbial resilience. As a preliminary observation, this finding warrants further investigation to validate the role of *Ligilactobacillus* enrichment and its potential gut microbiome-modulatory and neuroprotective relevance in ageing models.

*Ligilactobacillus* is a genus of lactic acid bacteria that has recently gained attention as a potential probiotic candidate [48]. Before March 2020, approximately 260 species were classified under the genus *Lactobacillus*. However, following a major taxonomic reclassification of the families *Lactobacillaceae* and *Leuconostocaceae*, the genus was divided into 25 new genera, resulting in the reclassification of *Lactobacillus salivarius* as *Ligilactobacillus salivarius* [49]. *Ligilactobacillus salivarius* is a Gram-positive, anaerobic rod commonly found in the intestines of mammals and birds [50]. Several in vitro and in vivo studies have demonstrated its potential anti-inflammatory properties, including the upregulation of anti-inflammatory cytokines such as IL-10 and downregulation of pro-inflammatory cytokines such as TNF-α, IL-6, and IL-1β [51,52]. Similarly, studies by Lv et al. [53] and Shi et al. [54] reported that *Ligilactobacillus salivarius* Li01, isolated from healthy human faeces, helped protect intestinal barrier integrity, reduce bacterial translocation and serum cytokine levels, and enhance gut microbial diversity. While the present findings tentatively suggest a similar enrichment pattern, further investigations are required to determine whether the increased *Ligilactobacillus* abundance observed here translates into measurable functional or anti-inflammatory outcomes.

The decrease in *Lachnospiraceae* and *Prevotellaceae* observed in both kefir-treated groups compared to the D-gal-treated group was not statistically significant but may indicate a potential trend towards reduced abundance. Nevertheless, this preliminary observation is in line with the findings of Van et al. [45], who reported that administration of kefir strains Fr1 and UK4 in mice led to a reduced prevalence of *Lachnospiraceae*. Members of the *Lachnospiraceae* family are part of the core gut microbiota and are among the main producers of SCFAs, particularly butyrate, an essential energy source for colonic epithelial cells. Butyrate contributes to shaping the gut microbial environment, strengthening the gut barrier, modulating host immunity, and potentially influencing brain function and behaviour via the gut–brain axis [55].

Although *Lachnospiraceae* are often considered beneficial for maintaining host gut and brain health, their effects appear to be complex and context-dependent. Different taxa within this family have been associated with both protective and pathogenic roles in various intraintestinal and extraintestinal disorders [55]. Some studies have linked reduced *Lachnospiraceae* abundance to neurodegenerative conditions, whereas others have reported certain *Lachnospiraceae* species promoting neuroinflammation and neurodegeneration through metabolite production. For instance, Nguyen et al. [56] reported increased *Lachnospiraceae* levels in patients with schizophrenia, while Zhang et al. [57] observed a decrease in the same family in a similar patient group. These inconsistencies underscore the need for further studies to clarify the context-specific functions of *Lachnospiraceae* and their relevance to gut–brain health.

*Prevotella*, a Gram-negative bacterium, exhibits both beneficial and detrimental effects in humans, depending on the strain. Beneficially, certain strains improve cardiovascular health and enhance glucose metabolism. However, others display pathobiontic properties, contributing to obesity, metabolic syndrome, inflammatory bowel disease, and various inflammatory conditions such as asthma, bacterial vaginitis, rheumatoid arthritis, and HIV infection. A higher abundance of *Prevotella* has also been associated with Alzheimer’s disease [58], Parkinson’s disease [59], and other inflammatory disorders [60], suggesting its potential role in promoting intestinal dysbiosis and inflammation. Iljazovic et al. [61] demonstrated that *Prevotella* disrupts the gut microbiome and decreases SCFA levels in gnotobiotic mice and experimental colitis models, thereby exacerbating intestinal inflammation. Likewise, Macia et al. [62] reported that colonisation with *Prevotella* reduced SCFA production in mice, which was associated with decreased colonic IL-18 levels, a cytokine crucial for maintaining intestinal homeostasis and defence against infection. Kim et al. [63] further showed that D-gal administration in 2,4-dinitrochlorobenzene-induced atopic dermatitis in BALB/c mice increased the relative abundance of *Prevotella* and *Ruminococcus*, while decreasing *Bacteroides* at the genus level. In addition, HS [58] reported that *Prevotella* interacts with dendritic cells and may contribute to Alzheimer’s disease development. *Prevotella*-associated gut microbiota changes can enhance the production of pro-inflammatory cytokines, leading to an inflammatory environment characterised by elevated TNF-α and IL-1 levels [58]. This heightened inflammation may drive neuroinflammation in the CNS, ultimately resulting in neuronal damage.

This preliminary study acknowledges several limitations that warrant careful consideration. The small sample size may have constrained statistical power, reducing the ability to detect subtle microbial shifts with confidence and increasing the likelihood that observed differences represent biological trends rather than definitive effects. Additionally, the absence of functional analyses such as PICRUSt2 prediction, LEfSe profiling, and SCFA quantification limits the interpretation of how specific microbial alterations translate into host physiological outcomes. Therefore, while the microbial patterns identified here provide valuable exploratory insights into kefir water’s modulatory potential, they should be interpreted with caution. Future investigations incorporating larger sample sizes and integrative metagenomic and metabolomic approaches will be essential to validate these preliminary findings and clarify the underlying functional mechanisms.

To further substantiate the biological relevance of these microbial trends and confirm the successful establishment of the ageing model, our related study (submitted as a separate manuscript) demonstrated that D-gal administration induced marked ageing-related physiological alterations, including elevated liver and kidney biochemical parameters (serum AST, ALT, urea, triglycerides, and LDL), increased serum pro-inflammatory cytokines (TNF-α, IL-2, IL-6, IL-β, and IFN-γ), and evident histopathological damage in the liver, spleen, frontal cortex, and hippocampus. These changes collectively validate the establishment of the D-gal-induced ageing model. Notably, both kefir water treatments markedly ameliorated these alterations, restoring biochemical, histopathological alterations, and inflammatory markers toward control levels. Transcriptomic and qPCR analyses further demonstrated regulation of key neuroinflammatory pathways, including chemokine, PI3K–AKT–mTOR, and MAPK signalling cascades in both kefir-treated groups, collectively supporting its systemic anti-inflammatory and neuroprotective potential. Neither D-gal nor kefir-treated groups significantly affected body weight or organ indices. Consistent with these findings, the current microbiome analysis showed that both kefir pre-treatment and co-treatment groups exhibited similar enrichment within the family *Lactobacillaceae*, particularly the genus *Ligilactobacillus*. This pattern suggests that either early or concurrent kefir administration may facilitate more stable colonisation of beneficial bacteria in the gut. Such microbial modulation could underlie the observed neuroprotective and anti-inflammatory effects of kefir water through gut–brain axis communication. Collectively, these findings highlight the importance of future investigations integrating functional, metagenomic, and metabolomic analyses to elucidate the mechanistic links between kefir-induced gut microbiota modulation and gut–brain axis-mediated neuroprotection.

## 5. Conclusions

Overall, kefir water treatments tended to enhance the abundance of beneficial taxa, such as *Muribaculaceae* (*Muribaculum*) and *Lactobacillaceae* (*Lactobacillus* and *Ligilactobacillus*), while reducing potential pathobiontic families, including *Lachnospiraceae* (*Lachnospiraceae* NK4A136 group) and *Prevotellaceae* (*Prevotellaceae* UCG-001). These compositional trends suggest a possible role of kefir water in supporting a healthier gut microbial community, although further studies are needed to determine the functional significance of these observations. The consistent enrichment of the family *Lactobacillaceae*, particularly the *Ligilactobacillus*, in the kefir-treated groups, suggests that either early or concurrent kefir administration may facilitate more stable colonisation of beneficial bacteria in the gut. Kefir co-treatment appeared to normalise the *Firmicutes*/*Bacteroidota* ratio disrupted by D-gal administration, suggesting a trend towards restoration of gut microbial balance. In contrast, kefir pre-treatment for 28 days was associated with a lower *Firmicutes*/*Bacteroidota* ratio, reflecting a distinct modulatory pattern that warrants further investigation to determine its biological relevance.

While these findings did not reach statistical significance, likely due to the limited sample size and short duration of D-gal exposure, they indicate that kefir water may exert mild modulatory effects on gut microbial composition, potentially counteracting early signs of D-gal-induced dysbiosis. These preliminary trends underscore kefir water’s potential as a functional dietary intervention for maintaining gut microbial balance during ageing, though confirmation through studies with larger cohorts and longer treatment durations (8–12 weeks) is essential to substantiate these effects and clarify the underlying host–microbiota mechanisms.

## Figures and Tables

**Table 1:** Alpha diversity indices of gut microbiota in D-galactose-induced mice treated with kefir water.

Groups	Operational Taxonomic Units (OTUs)	Species Richness Abundance-Based Coverage Estimators (ACE)	Diversity Index		
			Chao1	Shannon	Simpson (I/D)
Control	359.50 ± 5.50	360.16 ± 5.88	362.50 ± 8.50	4.89 ± 0.07	62.56 ± 5.46
D-gal	338.50 ± 0.50	340.03 ± 0.03	340.00 ± 0.00	4.85 ± 0.03	68.06 ± 0.71
VE+D-gal	316.00 ± 33.00	316.47 ± 33.47	316.75 ± 33.75	4.63 ± 0.05	54.23 ± 1.02
pre-VE+D-gal	433.00 ± 2.00	435.28 ± 2.66	435.11 ± 2.44	5.09 ± 0.08	82.45 ± 10.45
Kefir+D-gal	281.00 ± 22.00	281.10 ± 22.10	281.00 ± 22.00	4.73 ± 0.12	57.19 ± 10.52
pre-Kefir+D-gal	308.50 ± 4.50	311.87 ± 5.68	315.17 ± 8.83	4.48 ± 0.07	46.33 ± 4.09

Data are presented as mean ± standard deviation (SD). All comparisons yielded *p* > 0.05 (Kruskal–Wallis test), indicating no statistically significant differences between groups. Control: normal mice without induced ageing; D-gal: negative control mice administered 500 mg/kg/day D-gal; Kefir: kefir water co-treated mice administered 10 mL/kg/day kefir water and 500 mg/kg/day D-gal; p.Kefir: kefir water pre-treated mice pre-administered 10 mL/kg/day kefir water before receiving 500 mg/kg/day D-gal; VE: vitamin E co-treated mice administered 200 mg/kg/day VE and 500 mg/kg/day D-gal; p.VE: vitamin E pre-treated mice pre-administered 200 mg/kg/day VE before receiving 500 mg/kg/day D-gal.

**Table 2:** Summary of Bacterial Taxa Relative Abundance at the Phylum Level.

Bacterial Phyla	Control	D-gal	Kefir	p.Kefir	VE	p.VE
*Bacteroidota*	0.620 ± 0.034	0.515 ± 0.101	0.620 ± 0.106	0.707 ± 0.064	0.663 ± 0.010	0.456 ± 0.043
*Firmicutes*	0.344 ± 0.029	0.448 ± 0.107	0.344 ± 0.096	0.263 ± 0.060	0.309 ± 0.001	0.499 ± 0.037
*Patescibacteria*	0.008 ± 0.002	0.010 ± 0.002	0.017 ± 0.006	0.013 ± 0.004	0.016 ± 0.005	0.005 ± 0.000
*Actinobacteriota*	0.006 ± 0.001	0.006 ± 0.000	0.014 ± 0.005	0.009 ± 0.003	0.006 ± 0.001	0.004 ± 0.001
*Campylobacterota*	0.009 ± 0.003	0.008 ± 0.003	0.001 ± 0.000	0.001 ± 0.000	0.003 ± 0.003	0.020 ± 0.005
*Desulfobacterota*	0.008 ± 0.001	0.004 ± 0.001	0.001 ± 0.000	0.000 ± 0.000	0.001 ± 0.001	0.011 ± 0.004
*Proteobacteria*	0.002 ± 0.001	0.002 ± 0.002	0.003 ± 0.001	0.006 ± 0.002	0.001 ± 0.000	0.002 ± 0.001
*Cyanobacteria*	0.002 ± 0.001	0.006 ± 0.004	0.000 ± 0.000	0.001 ± 0.001	0.000 ± 0.000	0.003 ± 0.002

Data are expressed as mean ± standard deviation (SD) from *n* = 2 per group. Statistical analysis was performed using the Kruskal–Wallis test, which indicated no significant differences among groups: *Bacteroidota* (*p* = 0.251), *Firmicutes* (*p* = 0.238), *Patescibacteria* (*p* = 0.189), *Actinobacteriota* (*p* = 0.144), *Campylobacterota* (*p* = 0.103), *Desulfobacterota* (*p* = 0.069), *Proteobacteria* (*p* = 0.354), and *Cyanobacteria* (*p* = 0.223). Control: normal mice without induced ageing; D-gal: negative control mice administered 500 mg/kg/day D-gal; Kefir: kefir water co-treated mice administered 10 mL/kg/day kefir water and 500 mg/kg/day D-gal; p.Kefir: kefir water pre-treated mice pre-administered 10 mL/kg/day kefir water before receiving 500 mg/kg/day D-gal; VE: vitamin E co-treated mice administered 200 mg/kg/day VE and 500 mg/kg/day D-gal; p.VE: vitamin E pre-treated mice pre-administered 200 mg/kg/day VE before receiving 500 mg/kg/day D-gal.

**Table 3:** Relative abundance (%) of the bacterial genera *Lactobacillus*, *Ligilactobacillus*, and HT002 across treatment groups.

Genus	Control	D-gal	Kefir	p.Kefir	VE	p.VE
*Lactobacillus*	63.0 ± 2.0	50.0 ± 1.0	38.5 ± 0.5	45.0 ± 15.0	51.0 ± 0	38.0 ± 3.0
*Ligilactobacillus*	8.0 ± 3.0	15.5 ± 2.5	29.5 ± 3.5	34.0 ± 14.0	13.5 ± 0.5	30.0 ± 6.0
HT002	24.0 ± 1.0	28.5 ± 1.5	23.0 ± 5.0	17.5 ± 1.5	29.5 ± 0.5	28.5 ± 0.5

Data are presented as mean ± standard deviation (SD). All comparisons yielded *p* > 0.05 (Kruskal–Wallis test), indicating no statistically significant differences between groups. Note: The data correspond to the Krona chart (Figure 6) and represent mean relative abundances calculated from all biological replicates (*n* = 2), providing an overall summary of group-level microbial composition.

## Data Availability

The raw data supporting the conclusions of this article will be made available by the authors on request.

## Supplementary Materials

The following supporting information can be downloaded at: https://www.mdpi.com/article/10.3390/foods14223851/s1, Table S1: Standard dietary composition of AIN-93G rodent diets; Table S2: Coverage estimators of gut microbiota in kefir water-treated D-galactose mice; Table S3: Calculated relative abundances of bacterial taxa at the family level across all treatment groups corresponding to the graphical data presented in Figure 4; Table S4: Calculated relative abundances of bacterial taxa at the genus level across all treatment groups corresponding to the graphical data presented in Figure 5; Figure S1: Rank abundance curve showing species richness and evenness across all experimental groups; Figure S2: Rarefaction curve showing observed species richness across all experimental groups as a function of sequencing depth; Figure S3: Relative abundance of bacterial taxa at the family level across all treated groups; Figure S4: Relative abundance of bacterial taxa at the genus level across all treated groups.
