# Supplementary material for: Preliminary Evaluation of the Gut Microbiota Modulatory Potential of Malaysian Kefir Water in Ageing Mice
Source: Foods. 2025 Nov 11;14(22):3851. doi: 10.3390/foods14223851 (PMC12651598; doi:10.3390/foods14223851)
Supplement: Supplementary file 1 [file foods-14-03851-s001.zip › foods-3926265-supplementary.pdf]

Supplementary Information (SI)

Table S1. Standard dietary composition of AIN-93G rodent diets.

**Ingredients**	**Dietary Composition (g/kg)**
Casein	145
Cellulose	50
Choline bitartrate	2.5
Corn starch	452.5
Dextrinized corn starch	132
L-cysteine	3
Mineral mix	35
Soybean oil	70
Sucrose	100
Vitamin mix	10
Total energy	3.83 kcal/kg

AIN, American Institute of Nutrition.

AIN-93G rodent diet (normolipidic and normocaloric) - composition recommended by the American Institute of Nutrition.

Table S2. Coverage estimators of gut microbiota in kefir water-treated D-galactose mice.

Sample ID	Reads	Filtered	Rarefied
S1	126245	125860	56725
S2	126939	126510	56725
S3	116751	116394	56725
S4	130470	130059	56725
S5	111187	110817	56725
S6	103838	103493	56725
S7	210887	150532	56725
S8	231517	165051	56725
S9	180287	128036	56725
S10	138025	98380	56725
S11	162998	115857	56725
S12	122094	121645	56725
Total	1761238	1492634	680700

Table S3. Calculated relative abundances of bacterial taxa at the family level across all treatment groups corresponding to the graphical data presented in Figure 4.

Sample	Control	D-gal	Kefir	p.Kefir	p.VE	VE
Muribaculaceae	0.340	0.257	0.350	0.499	0.235	0.479
Lachnospiraceae	0.177	0.260	0.126	0.055	0.374	0.080
Lactobacillaceae	0.059	0.107	0.133	0.129	0.014	0.151
Bacteroidaceae	0.137	0.103	0.144	0.099	0.113	0.089
Prevotellaceae	0.079	0.092	0.047	0.067	0.044	0.063

Table S4. Calculated relative abundances of bacterial taxa at the genus level across all treatment groups corresponding to the graphical data presented in Figure 5.

Sample	Control	D-gal	Kefir	p.Kefir	p.VE	VE
Bacteroides	0.137	0.103	0.144	0.099	0.113	0.089
Lachnospiraceae NK4A136 group	0.071	0.113	0.059	0.030	0.132	0.038
Prevotellaceae UCG-001	0.071	0.089	0.037	0.063	0.043	0.047
Lactobacillus	0.037	0.054	0.054	0.067	0.006	0.078
Ligilactobacillus	0.005	0.017	0.043	0.036	0.004	0.020
Muribaculum	0.016	0.008	0.009	0.019	0.008	0.018

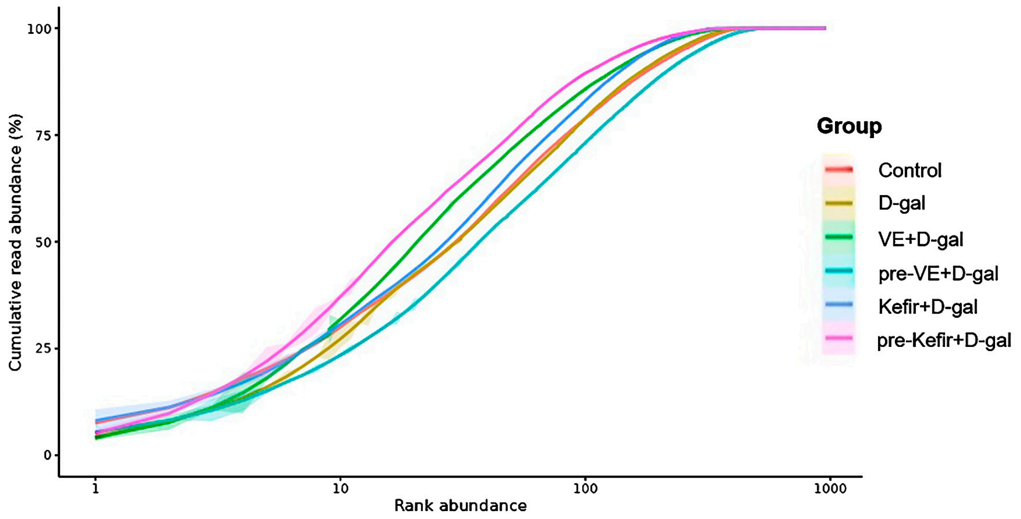

Figure S1. Rank abundance curve showing species richness and evenness across all experimental groups. The width of the curve along the x-axis represents species richness, while the slope of the curve indicates species evenness. This figure is provided to illustrate the distribution of species within each sample and to assess the coverage and reliability of the microbiome data. Control: normal mice without induced ageing; D-gal: negative control mice administered 500 mg/kg/day D-gal; Kefir: kefir water co-treated mice administered 10 mL/kg/day kefir water and 500 mg/kg/day D-gal; pre-Kefir: kefir water pre-treated mice pre-administered 10 mL/kg/day kefir water before receiving 500 mg/kg/day D-gal; VE: vitamin E co-treated mice administered 200 mg/kg/day VE and 500 mg/kg/day D-gal; pre-VE: vitamin E pre-treated mice pre-administered 200 mg/kg/day VE before receiving 500 mg/kg/day D-gal.

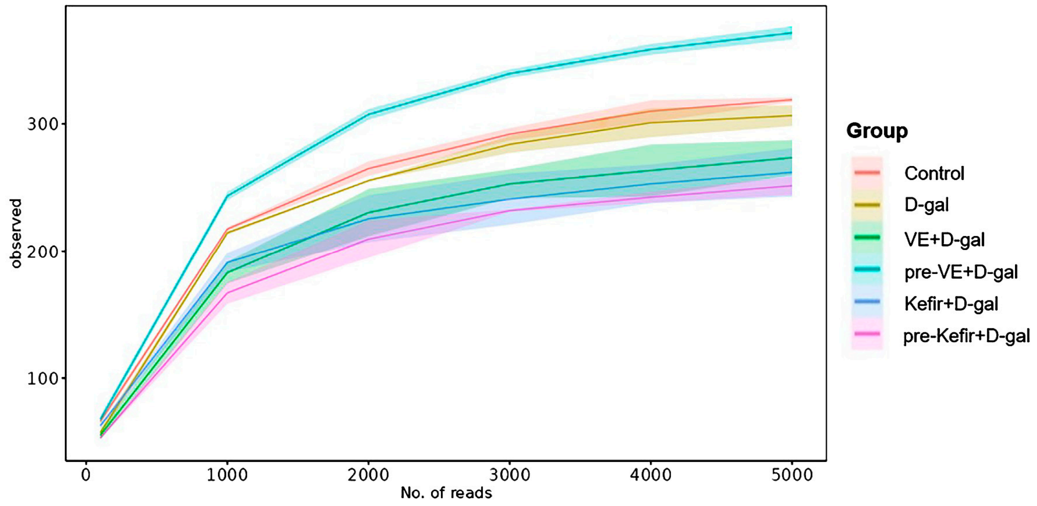

Figure S2. Rarefaction curve showing observed species richness across all experimental groups as a function of sequencing depth. The number of observed species is plotted on the y-axis, while the number of sequencing reads is on the x-axis. A higher curve indicates greater observed richness, reflecting a higher number of detected species at comparable sequencing depths. Curves that begin to plateau suggest that most species within the sample have been detected, and additional sequencing would likely reveal few new species. This analysis primarily evaluates sequencing reliability and coverage. Control: normal mice without induced ageing; D-gal: negative control mice administered 500 mg/kg/day D-gal; Kefir: kefir water co-treated mice administered 10 mL/kg/day kefir water and 500 mg/kg/day D-gal; pre-Kefir: kefir water pre-treated mice pre-administered 10 mL/kg/day kefir water before receiving 500 mg/kg/day D-gal; VE: vitamin E co-treated mice administered 200 mg/kg/day VE and 500 mg/kg/day D-gal; pre-VE: vitamin E pre-treated mice pre-administered 200 mg/kg/day VE before receiving 500 mg/kg/day D-gal.

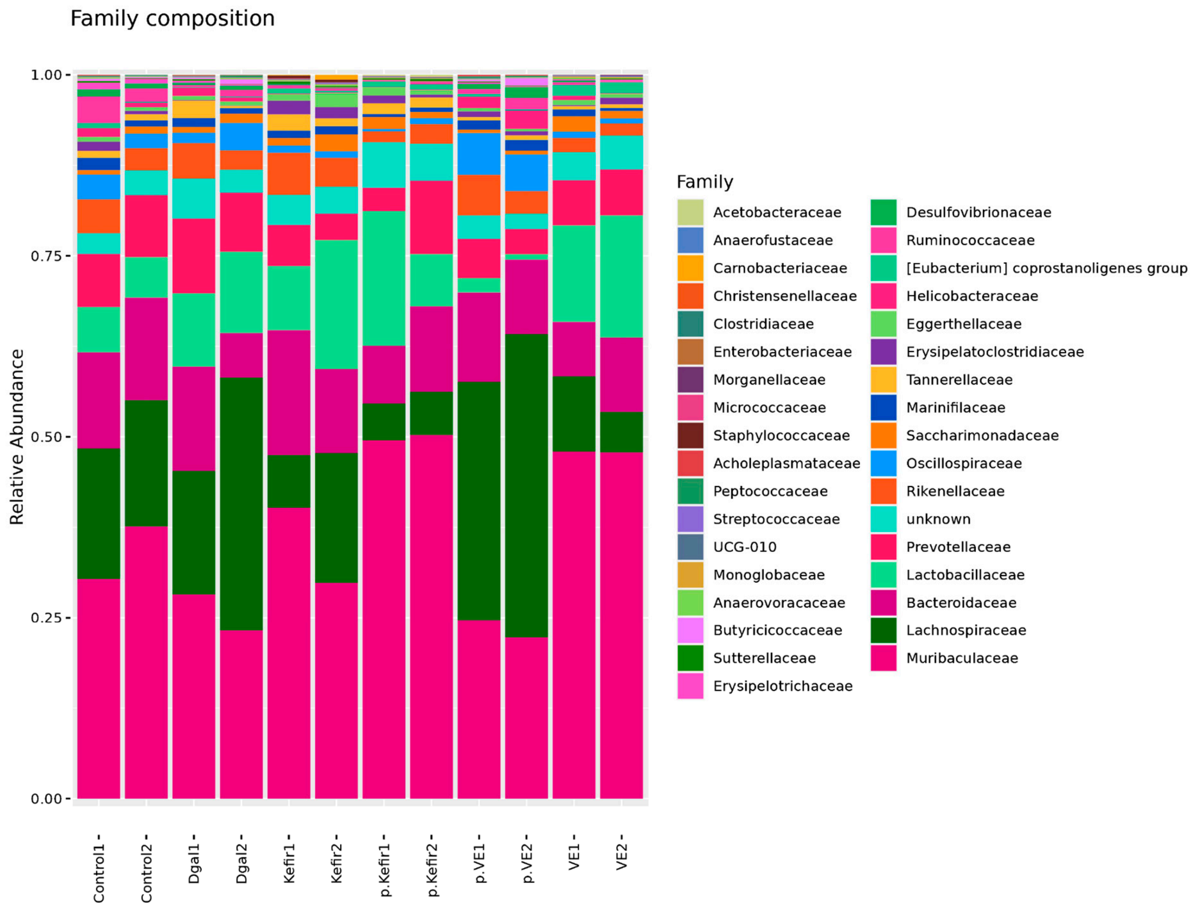

Figure S3. Relative abundance of bacterial taxa at the family level across all treated groups. Control: normal mice without induced ageing; D-gal: negative control mice administered 500 mg/kg/day D-gal; Kefir: kefir water co-treated mice administered 10 mL/kg/day kefir water and 500 mg/kg/day D-gal; p.Kefir: kefir water pre-treated mice pre-administered 10 mL/kg/day kefir water before receiving 500 mg/kg/day D-gal; VE: vitamin E co-treated mice administered 200 mg/kg/day VE and 500 mg/kg/day D-gal; p.VE: vitamin E pre-treated mice pre-administered 200 mg/kg/day VE before receiving 500 mg/kg/day D-gal.

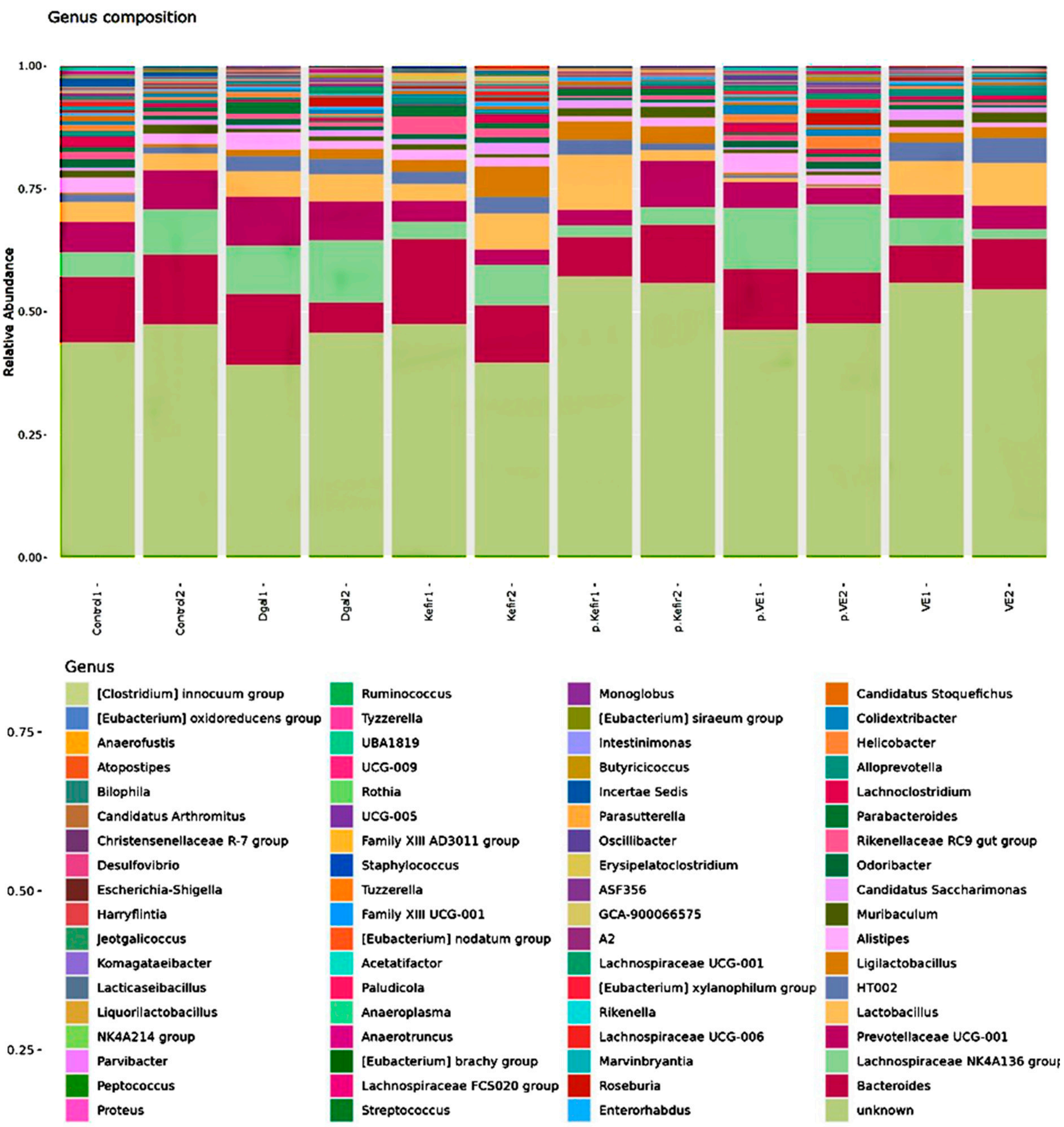

Figure S4. Relative abundance of bacterial taxa at the genus level across all treated groups. Control: normal mice without induced ageing; D-gal: negative control mice administered 500 mg/kg/day D-gal; Kefir: kefir water co-treated mice administered 10 mL/kg/day kefir water and 500 mg/kg/day D-gal; p.Kefir: kefir water pre-treated mice pre-administered 10 mL/kg/day kefir water before receiving 500 mg/kg/day D-gal; VE: vitamin E co-treated mice administered 200 mg/kg/day VE and 500 mg/kg/day D-gal; p.VE: vitamin E pre-treated mice pre-administered 200 mg/kg/day VE before receiving 500 mg/kg/day D-gal.
